# Maf deficiency in T cells dysregulates T_reg_ - T_H_17 balance leading to spontaneous colitis

**DOI:** 10.1038/s41598-019-42486-2

**Published:** 2019-04-16

**Authors:** Claire Imbratta, Marine M. Leblond, Hanifa Bouzourène, Daniel E. Speiser, Dominique Velin, Grégory Verdeil

**Affiliations:** 1 Department of Oncology, University of Lausanne, Lausanne, 1066 Switzerland; 2 UNISciences, University of Lausanne, UniLabs, Lausanne 1066 Switzerland; 3 Service of Gastroenterology and Hepatology, Department of Medicine, Lausanne University Hospital, Lausanne, 1066 Switzerland

## Abstract

The maintenance of homeostasis in the gut is a major challenge for the immune system. Here we demonstrate that the transcription factor MAF plays a central role in T cells for the prevention of gastro-intestinal inflammation. Conditional knock out mice lacking *Maf* in all T cells developed spontaneous late-onset colitis, correlating with a decrease of FOXP3^+^RORγt^+^ T cells proportion, dampened IL-10 production in the colon and an increase of inflammatory T_H_17 cells. Strikingly, FOXP3^+^ specific conditional knock out mice for MAF did not develop colitis and demonstrated normal levels of IL-10 in their colon, despite the incapacity of regulatory T cells lacking MAF to suppress colon inflammation in *Rag1*^−/−^ mice transferred with naïve CD4^+^ T cells. We showed that one of the cellular sources of IL-10 in the colon of these mice are T_H_17 cells. Thus, MAF is critically involved in the maintenance of the gut homeostasis by regulating the balance between T_reg_ and T_H_17 cells either at the level of their differentiation or through the modulation of their functions.

## Introduction

*Maf* encodes for a transcription factor belonging to the AP-1 family (MAF or c-MAF). It has been studied for many years in T cells, and more specifically in CD4^+^ T helper (T_H_) cell differentiation. Its role in *Il4* transcriptional regulation and T_H_2 regulation was first established using a transgenic mouse system for *Maf*^1,2^. *Maf* was also shown to play a prominent role in T_H_17 cells in both mice and human, through the regulation of *Il10* and *Il23r* expression^3,4^ and in T_FH_ cells together with the transcription factor *Bcl6*^5^. MAF inactivation in T cells affects susceptibility to disease in a context-specific manner, depending on the T cell polarization induced by this disease with a general tendency to increase inflammatory responses over tolerance^6^. In invariant natural killer T (iNKT) cells, MAF regulates the expression of IL-17A and RORγt^7^. Interestingly, *Maf* was found upregulated in CD8^+^ T cells infiltrated in human and murine melanoma. This expression led to intratumoral T cell dysfunction through the regulation of genes involved in T cell exhaustion^8^.

The potential role of *Maf* in regulatory T cells (T_reg_) is less clear. MAF cooperates with AhR in FOXP3^−^ T regulatory 1 (T_R1_) cells to control *Il10* expression^9^. Recently, a subset of T_reg_ expressing both FOXP3 and RORγt, the master transcription factor of T_H_17 cells, has been characterized in more detail^10^. Mostly present in the gut, RORγt^+^ T_reg_ have enhanced suppressive activity compared to RORγt^-^ T_reg_ and maintain gut homeostasis to microbiota^11–13^. The development of this population, described in both mice and human^11,12^, is tightly linked to the presence of the microbiota but not to dietary antigens^14^. Transcriptomic analysis showed an enriched expression of *Maf* in RORγt^+^ T_reg_^13^ and it was shown that the inactivation of *Maf* in T_reg_ strongly affects its function and differentiation into RORγt^+^ T_reg_^15,16^. Recent studies have linked MAF to the modulation of a large immunoregulatory and tissue-residency program in human T_H_17 cells producing Il-10. These results are reminiscent of the role of MAF in the regulation of tolerance in the gut that is dependent on induced Treg cells^17^. MAF can bind in the vicinity of many genes expressed in recently activated T_H_17 cell subsets. This study suggests that binding with other transcriptional partners to regulatory regions of the genome would explain the different effect of MAF on the expression of genes encoding either tolerogenic or inflammatory molecules.

In an attempt to clarify the role of *Maf* in T cells *in vivo*, we studied mice inactivated for *Maf* in all T cells (*Maf*^*ΔTcells*^). Interestingly, these mice developed late onset colitis correlating with a decrease of RORγt^+^ T_reg_ and an increase of T_H_17 cells in the colon and the mesenteric LNs. The disease was associated with increased production of TNFα, IFNγ, IL-1β and a lack of IL-10 in the colon of *Maf*^*ΔTcells*^ mice. Using an adoptive cell transfer model in *Rag1*^−/−^ mice, we showed that *Maf*-deficient T_reg_ are inefficient in preventing colitis, demonstrating the role of *Maf* in regulating T_reg_ function. Moreover, we observed that *Maf*^*ΔTcells*^ mice develop exacerbated T_H_17 response against *Helicobacter pylori*, a human pathobiont colonizing the stomach mucosa, leading to the inability of the bacteria to establish a chronic state of infection in *Maf*^*ΔTcells*^ mice. Strikingly, mice deficient for *Maf* in T_reg_ alone (*Maf*^*ΔTreg*^ mice) did not develop colitis and demonstrated normal levels of IL-10 in their colon. Compared to *Maf*^*ΔTcells*^, we observed that T_H_17 cells of *Maf*^*ΔTreg*^ mice produce IL-10. Taken together, our data demonstrated that *Maf* is playing a major role in the maintenance of gastro-intestinal homeostasis through the regulation of functions of both T_reg_ and T_H_17 cells. *Maf* regulates the differentiation of RORγt^+^ T_reg_, the suppressive activities of T_reg_ as well as the activity of T_H_17 cells from the gut.

## Results

### T cell specific Maf-deficient mice develop spontaneous colitis

**Figure 1:**
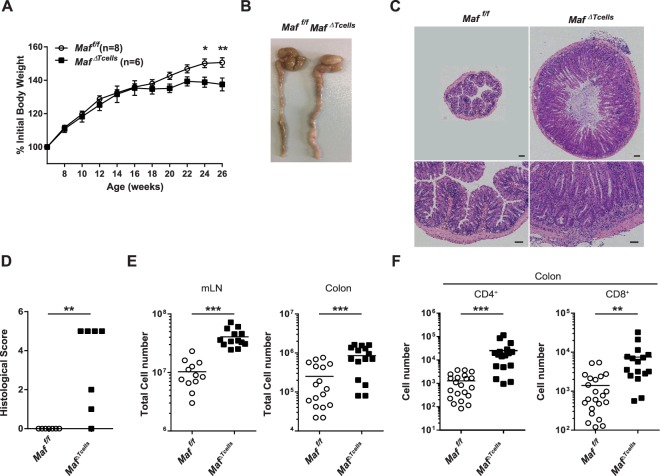
*Maf*^*ΔTcells*^ mice develop late-onset spontaneous colitis. (**A**) Body weight curves of *Maf*^*f/f*^ (n = 8) and *Maf*^*ΔTcells*^ (n = 6) male littermates. Statistical significances were calculated by a Bonferroni test after a significant two-way ANOVA. (**B**) Representative pictures of colons from *Maf*^*f/f*^ and *Maf*^*ΔTcells*^ mice. (**C**) Haematoxylin and eosin-stained sections of colons (upper picture: scale bar 200 µm; bottom pictures: scale bar 100 µm) and **(D**) histological scores (from 0 to 6) of microscopic changes in the colon from *Maf*^*f/f*^ and *Maf*^*ΔTcells*^ mice (n = 7 per group). **(E)** Total immune cell numbers in mLN (left) and colon (right) of *Maf*^*f/f*^ and *Maf*^*ΔTcells*^ mice (n > 10 per group). (**F**) Absolute cell numbers of CD4^+^ and CD8^+^ T cells in colons of *Maf*^*f/f*^ and *Maf*^*ΔTcells*^ mice (n > 10 per group). **(D**–**F)** Each symbol represents an individual mouse. **(E**,**F)** Data are representative of at least 2 independent experiments with at least 4 mice per group. All mice were over 20 weeks old. All graphs indicate means. Error bars display Standard Error Mean (SEM).

To study the role of *Maf* in T cells, we generated *CD4*^*cre*^ *Maf*^*fl/fl*^ mice (*Maf*^*ΔTcells*^), in which all T cells (CD4^+^ and CD8^+^) are inactivated for the expression of *Maf*. Interestingly, these mice spontaneously developed strong colitis, associated with a defect in weight gain starting at 16 weeks of age (Fig. 1A), concomitant with the appearance of diarrhea. These symptoms increased as the mice got older – with up to a 15% difference of body weight at 26 weeks of age between *Maf*^*ΔTcells*^ mice and Cre-negative littermates (*Maf*^*f/f*^) (Fig. 1A). Macroscopic and microscopic examination of the gastrointestinal system revealed severe inflammation of all the parts of the colon with a clear enlargement in most of the mice (Fig. 1B,C) but no clear difference in the length of the colon (Supplementary Fig. 1A). We did not observe similar features in other organs such as liver or kidney (Supplementary Fig. 1B-D). Hematoxylin and eosin staining on histological sections of colon samples showed massive infiltration of cells in the lamina propria and submucosal spread (Fig. 1C). The scoring of histological sections revealed that the majority of mice had severe colitis (4/7 mice with the score of 5) (Fig. 1D). We found a significant increase in the number of total immune cells both in the mesenteric LN (mLN) and in the colon of *Maf*^*ΔTcells*^ mice compared to the one from *Maf*^*f/f*^ littermates (Fig. 1E). Both CD4^+^ and CD8^+^ T cells were more abundant in the colon of *Maf*^*ΔTcells*^ mice compared to *Maf*^*f/f*^ littermates (Fig. 1F). *Maf*^*ΔTcells*^ mice treated with antibiotics developed no or only mild colitis (Supplementary Fig. 1E,F) demonstrating the requirement of microbiota in the development of this phenotype. Thus, the absence of *Maf* in T cells leads to spontaneous colitis in mice.

### *In vitro* differentiation of Maf-deficient CD4^+^ T cells in RORγt^+^ T_reg_ is defective

**Figure 2:**
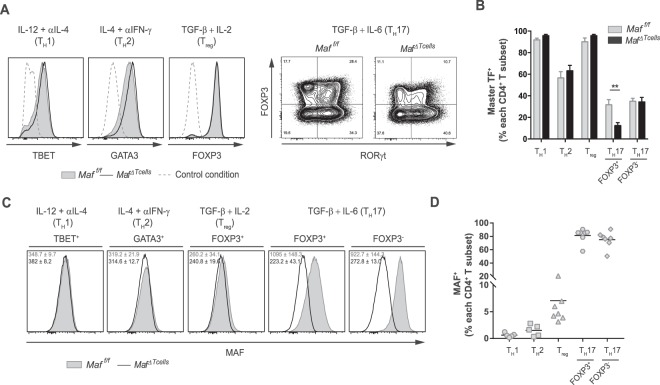
*In vitro* differentiation of *Maf*-deficient CD4^+^ T cells in RORγt^+^ T_reg_ is defective. CD4^+^ T cells isolated from splenocytes of *Maf*^*f/f*^ and *Maf*^*ΔTcells*^ mice were cultured for 5 days in T_H_1 (IL-12 + anti-IL-4), T_H_2 (IL-4 + anti-IFN-γ), T_reg_ (TGF-β +IL-2) or T_H_17 (TGF-β +IL-6) polarizing conditions. (**A**) Representative histograms for the expression of master transcription factors associated with each helper subset: TBET (T_H_1), GATA3 (T_H_2), FOXP3 (T_reg_) and RORγt (T_H_17 FOXP3^+^ and FOXP3^-^) from *Maf*^*f/f*^ and *Maf*^*ΔTcells*^ mice. (**B**) Percentage of each master transcription factor according to the condition of polarization among CD4^+^ T cells (**C**) Representative histograms for the expression of MAF in *in vitro* differentiated CD4^+^ T cell subsets according to the expression of their master transcription factor from *Maf*^*f/f*^ and *Maf*^*ΔTcells*^ mice. MFI is indicated for T cells from *Maf*^*f/f*^ (grey) or *Maf*^*ΔTcells*^ (black) mice. (**D**) Percentage of MAF^+^ cells among each T_H_ subset according to the expression of their master transcription factor from WT mice. Each symbol represents an individual mouse. Data are representative of 2 independent experiments with at least 2 mice per group. All graphs indicate means. Error bars display SEM.

As previously described, MAF has been shown to regulate cytokine production in various CD4^+^ T_H_ subsets. To determine whether MAF deficiency in CD4^+^ T cells can also alter the differentiation of any of these subsets, we differentiated naïve CD4^+^ T cells with polarizing medium to obtain T_H_1, T_H_2, T_reg_ and T_H_17 (FOXP3^−^ or FOXP3^+^) cells *in vitro* (Supplementary Fig. 2A–C). Comparison of the expression of the master transcription factors TBET (for T_H_1), GATA3 (for T_H_2), FOXP3 (for T_reg_) and RORγt (for T_H_17) between CD4^+^ T cells from *Maf*^*ΔTcells*^ and *Maf*^*f/f*^ mice did not show significant differences (Fig. 2A,B). However, in the T_H_17 polarizing condition, we observed the development of two distinct populations when analyzing FOXP3 expression. For the FOXP3^+^ population, also named RORγt^+^ T_reg_, there was a significant decrease in RORγt level in CD4^+^ T cells from *Maf*^*ΔTcells*^ mice compared to the one from *Maf*^*f/f*^ mice, from 35% to 10% of CD4^+^ T cells respectively (Fig. 2A,B). In these polarizing conditions, MAF levels were the highest in RORγt^+^ T_reg_ and T_H_17, with approximately 80% of MAF- expressing cells in both subsets. MAF is also expressed in RORγt^-^ T_reg_, though at lower level - around 8% of the cells - whereas it is almost absent in T_H_1 and T_H_2 (Fig. 2C,D). These *in vitro* experiments of differentiation suggest that MAF plays a role for the differentiation of RORγt^+^ T_reg_ and might impact the physiological functions of T_H_17 cells.

### T cell specific Maf-deficient mice are deprived of RORγt^+^ T_reg_

**Figure 3:**
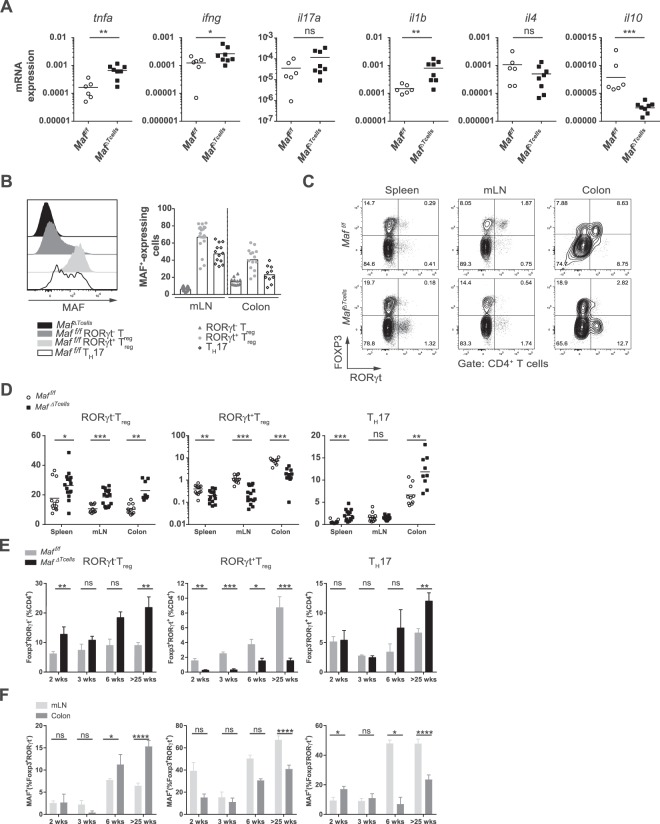
*Maf*^*ΔTcells*^ mice are deprived of RORγt^+^ T_reg_. **(A)** Quantitative RT–PCR of colonic tissues from *Maf*^*f/f*^ (n = 6) and *Maf*^*ΔTcells*^ (n = 8) mice for the indicated transcripts. Gene expression levels were normalized to *Beta2microglobulin*. **(B)** Representative histogram of the expression of MAF in mLN (left) and percentage of MAF^+^ cells (right) in mLN and colon in RORγt^−^ T_reg_, RORγt^+^ T_reg_ and T_H_17 cells from *Maf*^*f/f*^ mice (n > 10). **(C)** Representative contour plot of the expression of FOXP3 and RORγt in CD4^+^ T cells from spleens, mLN and colons of *Maf*^*f/f*^ and *Maf*^*ΔTcells*^ mice. **(D)** Percentage of RORγt^−^ T_reg_, RORγt^+^ T_reg_ and T_H_17 cells from spleens, mLN and colons of *Maf*^*f/f*^ and *Maf*^*ΔTcells*^ mice (n > 10 per group). **(E)** Percentage of RORγt^−^ T_reg_, RORγt^+^ T_reg_ and T_H_17 cells in CD4^+^ T cells from colons of *Maf*^*f/f*^ and *Maf*^*ΔTcells*^ mice at indicated ages (n > 4 per group). (**F**) Percentage of MAF^+^ cells among RORγt^−^ T_reg_, RORγt^+^ T_reg_ and T_H_17 cells from mLN and colons of *Maf*^*f/f*^ and *Maf*^*ΔTcells*^ mice at indicated ages (n > 4 per group). **(A**–**C)** Each symbol represents an individual mouse. Data are representative of at least 2 independent experiments with at least 3 mice per group. **(E**,**F**) Data are representative of at least 2 independent experiments with at least 2 mice per group. All graphs indicate means. Error bars display SEM.

To confirm the results obtained *in vitro*, we first looked at the abundance of transcripts encoding for cytokines in the total colon of aged *Maf*^*f/f*^ or *Maf*^*ΔTcells*^ mice (20 weeks or more). We found higher expression of *Tnfa, Ifng and Il1b* in *Maf*^*ΔTcells*^ mice compared to *Maf*^*f/f*^ littermates (Fig. 3A), in accordance with a higher infiltration of immune cells and a stronger inflammation in the colon. The transcripts encoding for *Il4* were slightly less abundant in *Maf*^*ΔTcells*^ mice and, strikingly, *Il10* expression level was much lower in colons from *Maf*^*ΔTcells*^ mice (Fig. 3A). We then determined the *in vivo* expression of MAF in the various subsets. MAF was expressed in RORγt^+^ FOXP3^+^ T_reg_ and RORγt^+^ FOXP3^−^ T_H_17 cells- around 40% and 20%, respectively - from the colon (Fig. 3B). In the colon, around 15% of RORγt^−^ T_reg_ expressed MAF (Fig. 3B). The proportion of RORγt^+^ T_reg_ was dramatically decreased in spleens, mLN and colons of *Maf*^*ΔTcells*^ mice, while we found a significant increase in percentages as well as total numbers of RORγt^−^ T_reg_ from these organs compared to *Maf*^*f/f*^ mice (Fig. 3C,D and Supplementary Fig. 3A). We also observed elevated percentages and cell numbers of T_H_17 cells in spleens, mLN and colons of *Maf*^*ΔTcells*^ mice compared to *Maf*^*f/f*^ mice (Fig. 3C,D and Supplementary Fig. 3A). In *Maf*^*f/f*^ mice, RORγt^+^ T_reg_ proportions increased with age to reach 10% of the total CD4^+^ T cell population in the colon at 25-weeks age mice (Fig. 3E). The defect in RORγt^+^ T_reg_ in *Maf*^*ΔTcells*^ mice is already detected at 2 weeks of age (i.e. the weaning period) compared to *Maf*^*f/f*^ mice (Fig. 3E). Interestingly, the level of MAF in these three populations followed the same upward trend with time (Fig. 3F). RORγt^−^ T_reg_ increase was already present in *Maf*^*ΔTcells*^ mice at the age of 2 weeks and tended to expand over time (Fig. 3E). However, the increase of T_H_17 cells was only observed at an advanced age in *Maf*^*ΔTcells*^ mice, correlating with the development of colitis. These data indicate that the deletion of MAF in T cells alters differentiation of highly suppressive RORγt^+^ T_reg_ whereas it favors RORγt^−^ T_reg_ accumulation and later T_H_17 cells expansion. Loss of the equilibrium between these subsets in the colon is associated with colitis onset.

### Maf-deficient T_reg_ fail to prevent colitis development *in vivo*

**Figure 4:**
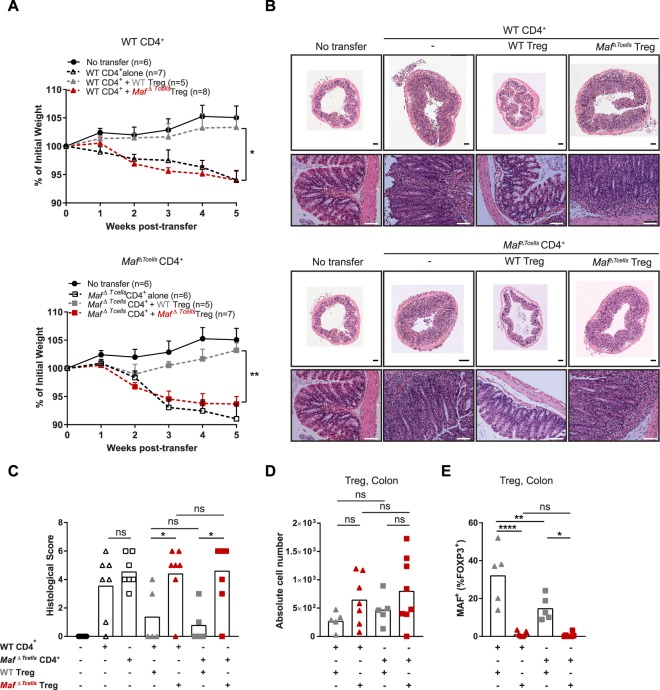
*Maf*-deficient T_reg_ fail to prevent colitis *in vivo*. CD4^+^CD45RB^hi^CD25^lo^ cells (0.5 × 10^6^) were intravenously injected alone or in combination with CD4^+^CD45RB^lo^CD25^hi^ cells (0.4 × 10^6^) into males and females *Rag1*^−/−^ mice from 10 to 15 weeks age. (**A**) Body weight curve of *Rag1*^−/−^ mice injected with either WT (upper) or *Maf*^*ΔTcells*^ naïve CD4^+^ T cells (bottom) alone or in combination with WT or *Maf*^*ΔTcells*^ T_reg_. Statistical significances were calculated by a Bonferroni test after a significant two-way ANOVA. (**B**) Haematoxylin and eosin-stained sections of colons (Upper picture: scale bar, 200 µm. Bottom pictures: scale bar, 100 µm) and **(C)** Histological scores (from 0 to 6) for microscopic changes in the colons of indicated groups of mice. (**D**) Absolute cell numbers of total FOXP3^+^ T cells in the colons of the indicated groups of mice (n > 5 per group). (**E**) Percentage of MAF^+^ cells among FOXP3^+^ T cells in the colons of the indicated groups of mice (n > 5 per group). (**A,C,D,E**) All results are representative of 2 independent experiments with at least 2 mice per group. (**C–E**), Each symbol represents an individual mouse. All graphs indicate means. Error bars display SEM.

The dramatic decrease in RORγt^+^ T_reg_ was associated with an increase in classical RORγt^−^ T_reg_ in *Maf*^*ΔTcells*^ mice (Fig. 3D). However, the development of spontaneous colitis in *Maf*^*ΔTcells*^ mice implies that *Maf*-deficient T_reg_ are unable to offset the decrease in highly suppressive RORγt^+^ T_reg_. This would suggest that the remaining *Maf*-deficient T_reg_ are dysfunctional and thus inefficient at suppressing T_H_17 cells. To test this hypothesis, we examined the capacity of *wild type (WT)* or *Maf*-deficient T_reg_ to suppress gut inflammation driven by the injection of naïve CD4^+^ T cells into *Rag1*^−/−^ mice^18^. We transferred either WT or *Maf*-deficient naïve CD4^+^ T cells alone or in the presence of either WT or *Maf*-deficient T_reg_ into *Rag1*^−/−^ recipient mice. Both WT and *Maf*-deficient naïve CD4^+^ T cells induced weight loss and cell infiltration in the colon when transferred alone (Fig. 4A,B) without any significant difference in the histologic score between the two groups (Fig. 4C). When we co-transferred WT T_reg_, the weight loss and the infiltration of cells in the colon were prevented with WT and *Maf*-deficient naïve CD4^+^ T cells (Fig. 4A,B). Strikingly, *Maf-*deficient T_reg_ failed to prevent colitis in all transferred groups compared to WT T_reg_, with a significant difference for the weight loss and the histological score (Fig. 4A–C). Histological sections of colons from recipient mice transferred with *Maf*-deficient T_reg_ displayed massive infiltration of cells in the associated lamina propria, with a disorganized architecture that was not observed in mice transferred with WT T_reg_ (Fig. 4B,C). We confirmed the presence of T_reg_ in all co-transferred groups (Fig. 4D) and the expression of MAF in WT transferred T_reg_ (Fig. 4E). Taken together, these results demonstrated that *Maf*-deficient T_reg_ are at least partially dysfunctional since they cannot prevent inflammation and colitis development.

### T_reg_ specific Maf-deficient mice do not develop spontaneous colitis

**Figure 5:**
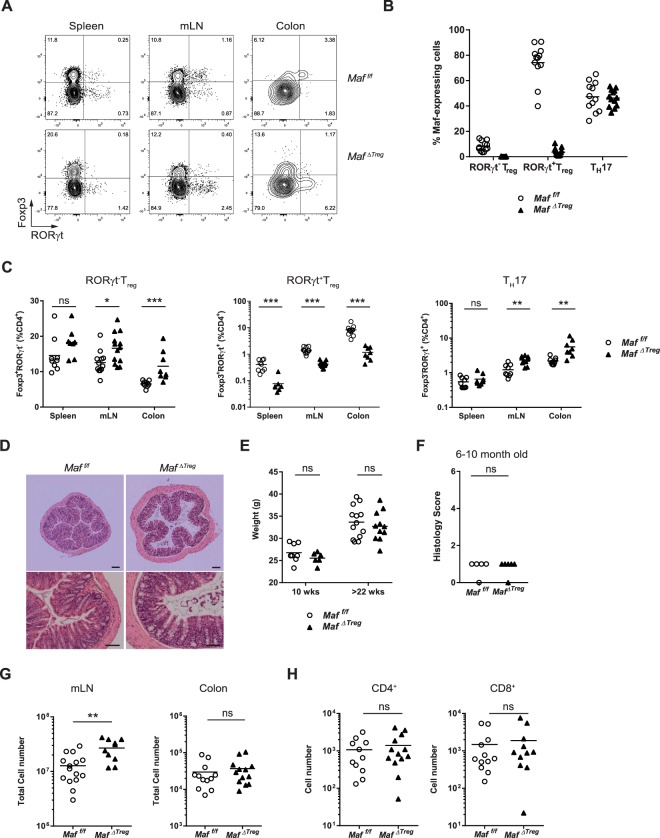
*Maf*^*ΔTreg*^ mice do not develop colitis. (**A**) Representative contour plot of the expression of FOXP3 and RORγt in CD4^+^ T cells from spleens, mLN and colons of *Maf*^*f/f*^ and *Maf*^*ΔTreg*^ mice. (**B**) Percentages of RORγt^-^ T_reg_, RORγt^+^ T_reg_ and T_H_17 cells from spleens, mLN and colons of *Maf*^*f/f*^ and *Maf*^*ΔTreg*^ mice (n > 8 per group). **(C)** Percentage of MAF^+^ cells among RORγt^-^ T_reg_, RORγt^+^ T_reg_ and T_H_17 cells from mLN of *Maf*^*f/f*^ and *Maf*^*ΔTcells*^ (n > 10 per group). **(D)** Haematoxylin and eosin-stained sections of colons from *Maf*^*f/f*^ and *Maf*^*ΔTreg*^ mice (Upper picture: scale bar, 200 µm; bottom pictures: scale bar, 100 µm). **(E)** Histological scores (from 0 to 6) of microscopic changes in the colon from *Maf*^*f/f*^ and *Maf*^*ΔTcells*^ mice at the age of 6 to 10 months (n = 5 per group). **(F)** Body weight of *Maf*^*f/f*^ and *Maf*^*ΔTreg*^ male mice of 10 and 22 weeks old (n > 6 per group). (**G**) Total cell numbers in mLN (left) and colons (right) of *Maf*^*f/f*^ and *Maf*^*ΔTreg*^ mice (n > 10 per group). (**H**) Absolute cell numbers of CD4^+^ and CD8^+^ T cells in colons of *Maf*^*f/f*^ and *Maf*^*ΔTreg*^ mice (n > 10 per group). (**B,C,E,H**) Each symbol represents an individual mouse. (**B,C,G,H**) Data are representative of at least 2 independent experiments with at least 4 mice per group. All mice were over 20 weeks old. All graphs indicate means.

We showed that both T_reg_ differentiation and function were largely affected in *Maf*^*ΔTcells*^ mice (Figs 3 and 4). To test whether the defect of MAF in Treg was sufficient to recapitulate the phenotype of colitis, we generated *Foxp3*^*YFPcre*^ *Maf*^*fl/fl*^ mice (*Maf*^*ΔTreg*^), in which only T_reg_ are inactivated for the expression of *Maf*. We observed a strong decrease in percentages as well as total numbers of RORγt^+^ T_reg_ in the colon and in the mLN, mirrored by a slight increase in percentages of RORγt^−^ T_reg_ and T_H_17 cells (Fig. 5A,C and Supplementary Fig. 3). We confirmed the complete deletion of *Maf* only in T_reg_ from *Maf*^*ΔTreg*^ mice (Fig. 5B). However, we did not observe any signs of colitis, confirmed by histological colon section and body weight measurement of these mice compared to *Maf*^*f/f*^ mice (Fig. 5D,E) even in aged (6–12 months) *Maf*^*ΔTreg*^ mice (Fig. 5F). Similarly, the number of immune cells infiltrated in the colon of *Maf*^*f/f*^ and *Maf*^*ΔTreg*^ was not different (Fig. 5G). We noticed a slight increase in cells in mLN (Fig. 5G) and no difference in the number of CD4^+^ or CD8^+^ T cells in the colon of *Maf*^*ΔTreg*^ mice compared to WT (Fig. 5H). This showed that *Maf* inactivation in T_reg_ is not sufficient to induce spontaneous colitis.

### Maf deletion impairs IL-10 production by T_reg_ and T_H_17 cells

**Figure 6:**
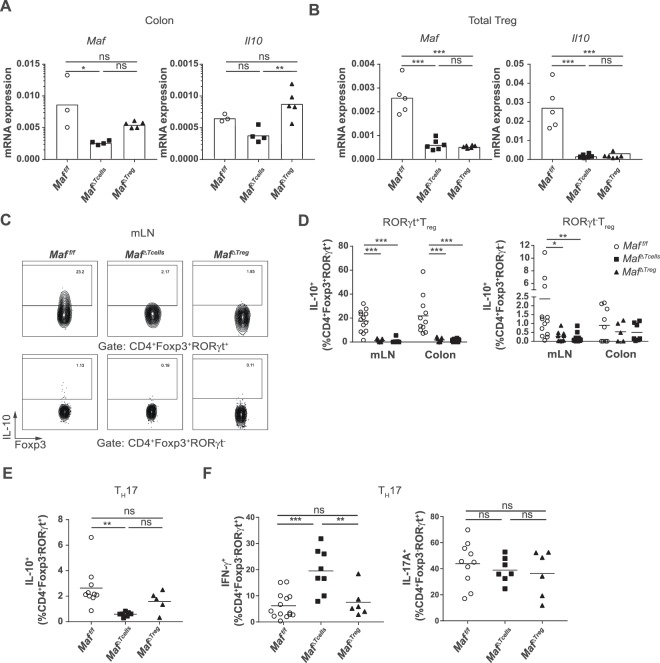
IL-10 expression is regulated by MAF in T_reg_ and T_H_17 cells. (**A**) Quantitative RT–PCR of colonic tissues from *Maf*^*f/f*^ (n = 3), *Maf*^*ΔTcells*^ (n = 4) and *Maf*^*ΔTreg*^ (n = 5) mice for the indicated transcripts. Gene expression levels were normalized to *Beta2microglobulin*. (**B**) Quantitative RT–PCR of isolated T_reg_ (CD4^+^CD25^+^CD45RB^lo^) of spleens and LN of *Maf*^*f/f*^ (n = 5), *Maf*^*ΔTcells*^ (n = 6) and *Maf*^*ΔTreg*^ (n = 6) mice for the indicated transcripts. Gene expression levels were normalized to *Beta2microglobulin*. (**C**) Representative contour plot of the expression of IL-10 and RORγt in T_reg_ from mLN of *Maf*^*f/f*^, *Maf*^*ΔTcells*^ and *Maf*^*ΔTreg*^ mice. (**D**) Percentage of IL-10^+^ cells among RORγt^-^ (left) and RORγt^+^ T_reg_ (right) in mLN and colon of *Maf*^*f/f*^, *Maf*^*ΔTcells*^ and *Maf*^*ΔTreg*^ mice after *ex vivo* restimulation (n > 8 per group). **(E)** Percentage of IL-10^+^ cells among T_H_17 cells from mLN of *Maf*^*f/f*^, *Maf*^*ΔTcells*^ and *Maf*^*ΔTreg*^ mice after *ex vivo* restimulation (n > 5 per group). **(F)** Percentage of IFN-γ^+^ and IL-17A^+^ cells among T_H_17 cells from colons of Cre-negative littermates, *Maf*^*ΔTcells*^ and *Maf*^*ΔTreg*^ mice after *ex vivo* restimulation (n > 6 per group). (**A,B,D–F**) Each symbol represents an individual mouse. Data are representative of 2 independent experiments. All mice were over 20 weeks old. All graphs indicate means. Statistical significances were calculated by a Tukey test after a significant one-way ANOVA.

To uncover why *Maf*^*ΔTreg*^ mice do not develop colitis, we measured transcript levels of cytokines encoding genes by quantitative RT-PCR in total colon of *Maf*^*f/f*^, *Maf*^*ΔTreg*^ or *Maf*^*ΔTcells*^ mice aged of more than 20 weeks. As expected, we found higher expression of transcripts encoding for *Maf* in colon from *Maf*^*ΔTreg*^ mice compared to colon from *Maf*^*ΔTcells*^ mice, confirming that conventional T cells infiltrated in the colon still expressed *Maf* in *Maf*^*ΔTreg*^ mice (Fig. 6A). When looking at transcripts encoding for *Il10*, we found a similar pattern of expression than the one for *Maf*, with a higher level of transcript in *Maf*^*f/f*^ and *Maf*^*ΔTreg*^ mice compared to *Maf*^*ΔTcells*^ mice (Fig. 6A). To confirm that T_reg_ from *Maf*^*ΔTreg*^ are really deficient for *Maf* and *Il10* production, T_reg_ from the spleen and LN of *Maf*^*f/f*^, *Maf*^*ΔTreg*^ or *Maf*^*ΔTcells*^ mice were sorted using flow cytometry. We measured high levels of transcripts for *Maf* and *Il10* in T_reg_ cells from *Maf*^*f/f*^ mice, while the levels of transcripts for these genes were low in T_reg_ cells from both *Maf*^*ΔTreg*^ and *Maf*^*ΔTcells*^ mice (Fig. 6B). Similarly, we confirmed that upon restimulation, T_reg_ from mLN and colon of *Maf*^*ΔTreg*^ or *Maf*^*ΔTcells*^ mice expressed decreased levels of IL-10 compared to *Maf*^*f/f*^ counterparts (Fig. 6C,D). This reduction is largely observed in RORγt^+^ T_reg_ but also to a lesser extent in RORγt^−^ T_reg_ from mLN of *Maf*^*ΔTreg*^ mice, suggesting a dependence for MAF for IL-10 production in T_reg_. These data suggest that *Maf* expression in FOXP3^−^ T cells is able to compensate for sufficient amounts of *Il10* production to prevent colitis development in *Maf*^*ΔTreg*^ mice. To determine which other T cell type could produce IL-10, we stimulated *ex-vivo* CD4^+^ T cells from mLN and colon of mice. We observed that IL-10 production by T_H_17 cells from mLN of *Maf*^*ΔTcells*^ mice was decreased compared to production from *Maf*^*f/f*^ and *Maf*^*ΔTreg*^ mice (Fig. 6E). This suggests that IL-10 production by T_H_17 cells in the gut is also regulated by *Maf*. The reduction of IL-10 expression not only by T_H_17 cells, but also T_reg_, could explain colitis onset observed only in mice where all the T cells are inactivated for *Maf*. Furthermore, we observed an increased percentage in IFNγ^+^T_H_17 cells while the percentage of IL-17A^+^ T_H_17 cells remained similar in *Maf*^*ΔTcells*^ mice compared to *Maf*^*f/f*^ or *Maf*^*ΔTreg*^ mice (Fig. 6F). Together, these data demonstrate that *Maf* is an essential driver of T_reg_ and T_H_17 immunoregulatory function, especially through IL-10 regulation.

### *Maf*^*ΔTcells*^ mice eliminate *Helicobacter pylori* through exacerbated T_H_17 response

**Figure 7:**
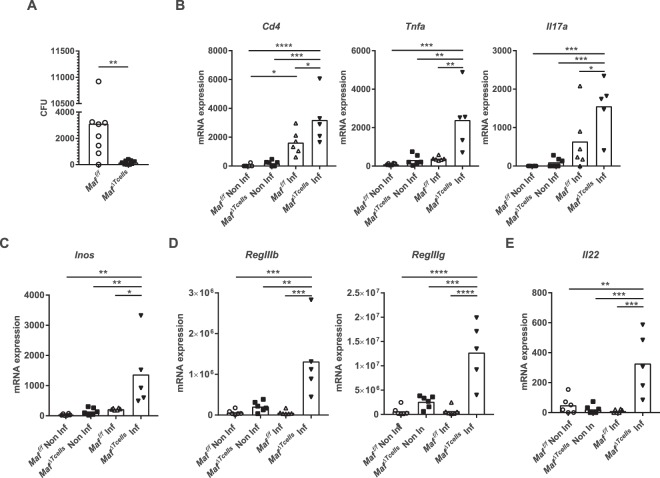
Elimination of Helicobacter pylori by *Maf*^*ΔTcells*^ mice through exacerbated T_H_17 response. (**A**) Numeration of CFU from stomachs of *Helicobacter pylori* infected *Maf*^*f/f*^ (n = 6) and *Maf*^*ΔTcells*^ (n = 5) mice. (**B–E**) Quantitative RT–PCR of stomachs from non-infected *Maf*^*f/f*^ (n = 6) and *Maf*^*ΔTcells*^ (n = 6) mice and *Helicobacter pylori* infected *Maf*^*f/f*^ (n = 6) and *Maf*^*ΔTcells*^ (n = 5) mice for the indicated transcripts. Gene expression levels were normalized to *Gapdh*. Each symbol represents an individual mouse. Data are representative of at least 2 independent experiments with at least 2 mice per group. Statistical significances were calculated by a Tukey test after a significant one-way ANOVA.

Recently, Gabrysova L *et al*. demonstrated that T_H_17 response was inhibited in *Maf*^*ΔTcells*^ mice in an experimental autoimmune encephalomyelitis (EAE) model^6^. This conclusion is not in line with our observation that *Maf*^*ΔTcells*^ mice develop a colitis (Fig. 1C) characterized by the accumulation of T_H_17 cells in the colon lamina propria (Fig. 3D). In order to re-evaluate our results in a different experimental setting, we infected *Maf*^*ΔTcells*^ mice with the human pathobiont *Helicobacter pylori*. *H. pylori* infects the stomach mucosa and relies on T_reg_ and IL-10 to chronically infect its host^19,20^. Moreover, it has been established that *H. pylori* can be cleared from the stomach mucosa by a T_H_17 response triggered by vaccination^21^. Two months after infection, quantification of the bacterial burden by numeration of colony-forming unit (CFU) in the mouse stomach showed that *Maf*^*ΔTcells*^ mice cannot be infected by *H. pylori* (Fig. 7A). Remarkably, as compared to infected *Maf*^*f/f*^ mice, we observed increased mRNA levels encoding for *Cd4*, *Tnfα*, *Inos*, *Il17*a, *Il22* and the antimicrobial peptides *RegIIIβ* and *RegIIIγ* in the stomach mucosa of *Maf*^*ΔTcells*^ mice (Fig. 7B–E). This pattern of increased expression, which is characteristic of a T_H_17 response, is very similar to the vaccine-induced T_H_17 response that clear *H. pylori* infection in WT mice^22^. In addition, we found higher *Il2* production as well as higher *Foxp3* level in *Maf*^*ΔTcells*^ mice (Supplementary Fig. 4). Collectively, these data suggest that *Maf*^*ΔTcells*^ mice can eliminate *H. pylori* infection by generating an exacerbated T_H_17 inflammatory program.

## Discussion

Our study shows that the deletion of *Maf* in all T cells (*Maf*^*ΔTcells*^ mice) drives spontaneous late-onset colitis (Fig. 1). To date, no study has shown that the deletion of *Maf* in T cells was sufficient and necessary to drive colitis. The development of the colitis is associated with the dysregulation of the T_reg_-T_H_17 equilibrium (Fig. 3D,E) and a large decrease in the production of IL-10 (Fig. 3A). Inactivation of *Maf* in T cells impaired the differentiation of RORγt^+^ T_reg_ (Fig. 3C) and favoured the accumulation of colitogenic T_H_17 cells in the colon of the mice (Fig. 3D). The onset of the colitis is dependent on the presence of microbiota since *Maf*^*ΔTcells*^ mice treated with antibiotics develop only mild colitis (Supplementary Fig. 1E,F). The first visible signs of colitis developed around the age of 15 weeks (Fig. 1A). It correlates with the natural differentiation and/or accumulation of RORγt^+^ T_reg_ and MAF expression in these cells in the colon of WT animals (Fig. 3E,F). This RORγt T_reg_ population is preferentially found in the colon (Fig. 3C) and represents the main producer of IL-10 (Fig. 6D)^10,16^. The role of MAF in the regulation of the RORγt T_reg_ population was demonstrated very recently using mice in which *Maf* is deleted in T_reg_ (*Maf*^*ΔTreg*^)^15,16^. In these two studies, the authors showed that the differentiation of this particular regulatory population is altered in the absence of *Maf*.

The low proportion of RORγt^+^ T_reg_ in the colon of *Maf*^*ΔTcells*^ mice was paradoxically associated with an increase in proportion and numbers of RORγt^−^ T_reg_. We provide evidence that T_reg_ in *Maf*^*ΔTcells*^ mice are not fully functional. Indeed, compared to WT T_reg_, *Maf*-deficient T_reg_ do not control the development of colitis induced by the transfer of naïve CD4^+^ T cells into *Rag1*^−/−^ mice (Fig. 4). Moreover, we observed that *Maf*-deficient RORγt^−^ T_reg_ produce lower level of IL-10 as compared to WT RORγt^−^ T_reg_ (Fig. 6C,D). These findings are in appearance not entirely in line with the study of Xu *et al*. showing that *Maf* -deficient nT_reg_ are still able to suppress colon inflammation in *Rag1*^−/−^ mice transferred with naïve CD4^+^ T cells^15^. However, given the nature of their experimental setting in which *Maf* -deficient nT_reg_ were isolated from *H. hepaticus* colonized mice and transferred in *H. hepaticus* infected mice, we assume that the antigen specificity of some *Maf*-deficient nT_reg_ could compensate for their partial loss of function. One other possibility would be the effect of potentially contaminating Maf-deficient T cells transferred with sorted Maf-deficient Treg. We assume that this potentially pathogenic T cells are also present in the “naïve” Maf-deficient T cells transferred in the mice. However, control Treg transferred together with “naïve” Maf-deficient T cells are still able to control colitis, which make this possibility unlikely. Altogether, it can be suggested that despite their normal differentiation, *Maf*-deficient RORγt^−^ T_reg_ are unable of preventing colitis due to their loss of function (decreased IL-10 production), combined to their low specificity to the gut microbiota in our experimental setting.

An impaired differentiation of colon RORγt^+^ T_reg_ was also observed in *Maf*^*ΔTreg*^ mice (Fig. 5A). Despite this absence, the mice did not develop colitis even at old ages (Fig. 5D–F). The absence of colitis onset is associated with a very limited accumulation of T_H_17 cells (Fig. 5C) and normal expression level of IL-10 in the colon (Fig. 6A). This result is similar to what is described previously in these mice, with only a mild inflammation detected in *H. hepaticus* free animals aged of 6 months^15^.

One of the differences between *Maf*^*ΔTreg*^ and *Maf*^*ΔTcells*^ mice is that RORγt^+^ T_H_17 cells do not express *Maf* in *Maf*^*ΔTcells*^ mice (Fig. 2C). It has been shown that MAF plays a prominent role in T_H_17 cells through the regulation of IL-10 production^4,17^. We indeed observed *in vivo* that RORγt^+^ T_H_17 cells from *Maf*^*ΔTcells*^ mice do not produce IL-10 while *Maf*^*f/f*^ and *Maf*^*ΔTreg*^ counterparts do (Fig. 6E). Furthermore, we showed an increase of IFN-γ^+^ T_H_17 cells from *Maf*^*ΔTcells*^ compared to *Maf*^*f/f*^ and *Maf*^*ΔTreg*^ mice (Fig. 6F).

*Maf* can affect other T cell subtypes that might participate to colitis development. A recent study on the role of MAF in iNKT cells showed that MAF inactivation in these cells leads to a decrease in IL-17a production^7^. In our system this would mean that inactivation of MAF in these cells would limit the development of colitis, which is however not the case. MAF regulates IL-4 but not IL-10 production in T_H_2 cells^23^. The absence of MAF leads to a decreased expression of Il-4^23^, which is not in accordance with an increased pathology. T_H_2 cells are a minority of the CD4 T cells that we find in the colon of the mice and do not increase among CD4 T cells in Maf^ΔTcells^ (Supplementary Fig. 3C). Despite the involvement of T_H_2 cells in the development of ulcerative colitis in human^24^, we think for these reasons that it is unlikely that the development of colitis after MAF inactivation is related to T_H_2 cells. T_R_1 cells, may also be a source of IL-10. Indeed, it was described that MAF is present in this population and regulates the expression of *Il10* together with AhR^9^. We did not find any IL-10 producing RORgt- Foxp3- CD4 T cells in the colon of our mice, which limits the potential role of Tr1 in our system (Supplementary Fig. 3C). Altogether, the absence of IL-10 production by both RORγt^+/−^ T_reg_ and T_H_17 cells associated with the increase production of IFN-γ by T_H_17 cells in *Maf*^*ΔTcells*^ mice most probably lead to colitis onset.IL-10 production by T_reg_ is essential to maintain homeostasis at environmental surfaces by directly suppressing pathogenic T_H_17 cells and T_H_17/T_H_1 cells^25^. Several years ago, work led by Rudensky *et al*. showed that selective disruption of *Il10* in T_reg_ leads to spontaneous colitis^26^. However, mice lacking IL10RA^27^ in T_reg_ that show reduced expression of IL-10, develop a more severe colitis, suggesting that compensatory mechanisms orchestrated by non-T_reg_ can participate to maintain homeostasis through regulation of T_reg_ function. Although restricted to the colon, the phenotype observed in our model looks similar to the colitis observed in T cell–specific IL-10 mutant mice^28^ or *Il10*^−/−^ mice^29^. This is in line with recent study showing that *Maf* is a common regulator of IL-10 in CD4^+^ T cells^6^. We were able to link this phenotype to decreased suppressive capacity of all *Maf*-deficient T_reg_, but particularly RORγt^+^ T_reg_, leading to uncontrolled T_H_17-driven inflammation.

Recent reports suggested that RORγt is induced after the differentiation of naïve cells into Foxp3^+^ T_reg_^13,30^. How MAF is regulating the development of these cells remains an open question. MAF is induced by TGF-β and IL-6 in CD4^+^ T_H_ subsets and CD8^+^ T cells^3,4,8,15^. Wheaton *et al*. proposed that IL-6 can upregulate MAF in pre-differentiated T_reg_ leading to RORγt acquisition. Indeed, RORγt has been identified as a direct target of MAF^6^. Observation that selective disruption of STAT3 in T_reg_ leads to a decrease of RORγt^+^ T_reg_ (11) in mice suggest that STAT3 may also be required in this process of differentiation. Thus, MAF appears to be at least in part regulated through STAT3 activating cytokines such as IL-6. However, what is exactly regulating MAF expression in the gut remains to be precisely determined.

A recent study showed that additionally to its direct regulation of *Il10* expression, *Maf* is also an inhibitor of *Il2*^6^. In the *Maf*-deficient T cells, the increased production of IL-2 has been shown to inhibit the T_H_17 cell response in the EAE induced model^6^. In our study, we observed that after infection with *H. pylori*, the bacteria is cleared from the stomach mucosa of *Maf*^*ΔTcells*^ mice but establish a chronic infection in *Maf*^*f/f*^ mice. We measured an increased level in transcripts coding for *Il2* and *Foxp3* in the stomach mucosa of *H. pylori*-infected *Maf*^*ΔTcells*^ mice (Supplementary Fig. 4), leading to the possibility that increased number of classical T_reg_ are recruited in the stomach mucosa of *Maf*^*ΔTcells*^ mice through an *Il2*-dependent pathway. However, we also found high amount of *Il17a* and *Il22*, indicating the recruitment of T_H_17 cells (Fig. 7B,E) leading to the clearance of *H. pylori* from the stomach mucosa of *Maf*^*ΔTcells*^ mice. These results clearly demonstrate that MAF plays a major role in maintaining the T_reg_ -T_H_17 equilibrium not only in the colon but also in the gastric mucosa. In addition, it can be suggested that the development of a vaccine directed against a pathobiont, such as *H. pylori*, might be facilitated by a vaccine formulation that prevent MAF expression in primed T cells.

Altogether, we established that the role of *Maf* was not only restricted to T_reg_ but also to conventional T cells, especially T_H_17 cells, establishing *Maf* as a major regulator of the T_reg_ – T_H_17 balance in the gastro-intestinal tract. On a broader perspective, MAF expression in colonic lymphocytes appears to be strongly associated with an anti-inflammatory type of response. However, the implication of *Maf* in immune responses appears to be largely location- and context-dependent^6^.

## Material and Methods

### Mice

*Rag1*^−/−^ and *Foxp3*^*YFPcre* 26^ mice were kindly provided by P.C Ho. *Foxp3*^*YFPcre*^ and *CD4*^*c*re^ ^31^ mice were crossed with *Maf*^*fl/fl*^ mice^32^ to generate *Foxp3*^*YFPcre*^ *Maf*^*fl/fl*^ (*Maf*^*ΔTreg*^) and *CD4*^*cre*^ *Maf*^*fl/fl*^ (*Maf*^*ΔTcells*^), respectively. Mice were bred and maintained in a specific-pathogen-free (SPF) environment, which excludes *Helicobacter hepaticus*, of the animal facility of the University of Lausanne. The animals used were cohoused and littermate controls are referred to as *Maf*^*f/f*^ mice. Experiments were performed in compliance with the University of Lausanne Institutional regulations and were approved by the veterinarian authorities of the Canton de Vaud (Switzerland).

### Isolation of cells

Single-cell suspensions from spleens and mesenteric lymph nodes were obtained after mashing the organs through a 70 µM cell strainer. Colons were collected in calcium and magnesium free Hank’s Balanced Salt Solution (HBSS) (ThermoFisher Scientific) supplemented with 2% of Fetal Calf Serum (FCS) (HBSS 2%) on ice. Samples were further flushed with HBSS 2% and cut longitudinally into 3–4 pieces (2–3 cm). Minced tissues were treated with EDTA 1 mM (ThermoFisher Scientific) and DTT 1 µM (AppliChem) in HBSS 10% solution for 20 min under shaking at 37 °C. After intraepithelial lymphocytes (IEL) removal, cells from the lamina propria were washed twice in HBSS 10% and incubated with Collagenase D (1 mg/ml) (Roche) and complete RPMI (ThermoFisher Scientific) for 30 min under shaking at 37 °C. To isolate leucocytes, supernatants were centrifuged in density gradients 40%/70% Percoll (GE Healthcare Life Sciences) for 30 min at 2000 rpm. All isolated cells were washed in complete RPMI and filtrated before staining.

### T cell isolation and transfer into *Rag1*^−/−^ mice

Donor cells were obtained from spleens and LN of both WT (obtained from Cre-negative littermates and C57Bl6 mice) and *Maf*^*ΔTcells*^ female mice. CD4^+^ T cells were enriched using EasySep Mouse CD4^+^ T Cell Isolation Kit (StemCell Technologies) according to the manufacturer’s recommendations. CD4^+^CD45RB^hi^CD25^lo^ and CD4^+^CD45RB^lo^CD25^hi^ were isolated by sorting on a FACS Aria cell sorter (BD Biosciences). CD4^+^CD45RB^hi^CD25^lo^ cells (0.5 × 10^6^) were intravenously injected alone or in combination with CD4^+^CD45RB^lo^CD25^hi^ cells (0.4 × 10^6^) into males and females *Rag1*^−/−^ mice from 10 to 15 weeks age. Mice were weighed and monitored weekly. 6 weeks after transfer, mice were sacrificed.

### Histopathology and scoring

Colons were fixed with histological tissue fixative (Sigma), embedded in paraffin and stained with haematoxylin and eosin. Histopathological score (0–6) was calculated based on inflammatory cells infiltration (0–3) and tissues abnormalities (0–3) by a pathologist.

### *In vitro* T cell differentiation

Naïve CD4^+^CD25^lo^CD62^hi^CD44^lo^ cells were isolated by using EasySep Mouse Naïve CD4^+^ T Cell Isolation Kit (Stemcell Technologies) from spleens and LN of *Maf*^*f/f*^ and *Maf*^*ΔTcells*^ mice, and activated with plate-bound anti-CD3 (Biolegend, 5 μg/ml) and soluble anti-CD28 (Biolegend, 1 μg/ml) supplemented with mIL12 (10 ng/ml) and neutralizing antibody anti-IL-4 (clone 11B11, 10 μg/ml) (T_H_1), mIL-4 (10 ng/ml) and anti-IFN-γ (clone: XMG-121,10 μg/ml) (T_H_2), hIL-2 (50U/ml) and TGF-β (10 ng/ml) (T_reg_), TGF-β (5 ng/ml) and IL-6 (40 ng/ml) (FOXP3^+^ and FOXP3^−^ T_H_17). Cells were incubated 5 days at 37 °C, 5% CO_2_.

### Antibodies, intracellular staining and flow cytometry

All antibodies used are listed in Table 1. For intracellular cytokine staining, cells were restimulated with PMA (Sigma, 50 ng/ml) and Ionomycin (Sigma, 100 ng/ml) for 4 h at 37 °C in presence of Golgi Plug (BD Biosciences). After staining for viability using LIVE/DEAD™ Fixable Aqua Dead Cell Stain Kit (ThermoFisher Scientific) and extracellular markers, cells were fixed and permeabilized with the FOXP3 Transcription Factor Staining Buffer Set (eBiosciences) according to manufacturer’s recommendations. Following permeabilization, cells were intracellularly stained for specific cytokine and acquired on a LSRII flow cytometer (BD). Data were analysed with FlowJo software V10.

**Table 1:** List of the antibodies used for flow cytometry.

Antibody	Clone	Reference	Fluoresence Labelling	Supplier	Dilution
CD25	PC61	20251–83	PE	eBioscience	1/3000
CD4	RM4–5	100549	BV711	Biolegend	1/100
CD45.2	104	109835	BV650	Biolegend	1/50
CD45RB	C363-16A	103307	PE	Biolegend	1/200
CD8	53–6.7	100743	BV605	Biolegend	1/500
C-MAF	sym0F1	50-9855-82	eFluor 660	eBioscience	1/100
FOXP3	FJK-16s	48-5773-82	eFluor 450	eBioscience	1/200
	MF-14	126406	Alexa Fluor 488	Biolegend	1/200
GATA-3	TWAJ	46-9966-42	PerCP-eFluor710	eBioscience	1/200
IFN-γ	XMG1.2	505826	PE/Cy7	Biolegend	1/200
IL-10	JES5-16E3	505007	PE	Biolegend	1/100
IL-17A	eBio17B7	53-7177-81	Alexa Fluor 488	eBioscience	1/200
RORγt	B2D	61-6981-82	PE-eFluor 610	eBioscience	1/200
T-BET	4B10	644814	APC	Biolegend	1/200

### Real-time qPCR analysis

Colons were collected, dried and ground into a powder using liquid nitrogen. Total RNA was isolated using RNAeasy Mini Kit (Qiagen, #74194) according to the manufacturer’s recommendations. cDNA was retro-transcribed using High-Capacity cDNA Reverse Transcription Kit (Applied Biosystems, # 4368814) and used for quantitative PCR. The KAPA SYBR® FAST qPCR Master Mix (2×) Kit (Sigma, #KK4618) was used for SYBR analysis. The housekeeping gene *beta2microglobulin* was used to normalize gene expression. Sequences of primers used are listed in Table 2.

**Table 2:** List of the primers used for quantitative RT-PCR.

Gene	Reverse (5′-3′)	Forward (5′-3′)
*beta2microglobulin*	AGACTGATACATACGCCTGCAG	GCAGGTTCAAATGAATCTTCAG
*ifnγ*	CAACAGCAAGGCGAAAAA	GGACCACTCGGATGAGCTC
*il10*	ACCTGCTCCACTGCCTTGCT	GGTTGCCAAGCCTTATCGGA
*il17a*	GCTCCAGAAGGCCCTCAGA	AGCTTTCCCTCCGCATTGA
*il1b*	TCGAGGCCTAATAGGCTCATCT	GCTGCTTCAGACACTTGCACAA
*il4*	GAAGCCCTACAGACGAGCTCA	GGGACGCCAT
*maf*	AACATATTCCATGGCCAGGG	GGATGGCTTCAGAACTGGCA
*tnfa*	TGGAAGTAGACAAGGTACAACCC	CATCTTCTCAAAATTCGAGTGACAA
*inos*	CATTGGAAGTGAAGCGTTTCG	CAGCTGGGCTGTACAAACCTT
*gapdh*	TCACCACCACCATGGAGAAGG	GCTAAGCAGTTGGTGGTCA
*regIIIb*	QT00239302 (Quiagen)	
*regIIIg*	QT00147455 (Quiagen)	
*foxp3*	QT00138369 (Quiagen)	

### Bacteria and infection

H. pylori P49, kindly provided by Harry Kleanthous (Acambis, Cambridge, MA), is a human clinical isolate adapted to mice. H. pylori P49 expresses VacA but not CagA. H. pylori P49 was grown on Helicobacter pylori-selective agar plate (Oxoid, Basingstoke, UK) and brain heart infusion broth supplemented with 0.25% yeast extract and 10% fetal calf serum (PAA, Pasching, Austria) under microaerophilic conditions, as previously describe^33^. Mice were treated on day 4 and 5 after birth with 5 × 10^8^ H. pylori P49, administered by orogastric gavage in 200 μL BHI.

### Antibiotics treatment

*Maf*^*f/f*^ and *Maf*^*ΔTcells*^ pregnant females were fed with antibiotics in their drinking water consisting of 0.5 mg/ml Amoxicillin (Mepha Pharma AG) and 5 mg/ml Enrofloxacine (Bayer). Antibiotic-containing drinking water was changed once a week until analysis. Offspring were then treated until their sacrifice at 17 weeks age.

### Statistical analysis

Unless otherwise stated, unpaired non-parametric Mann-Whitney t tests were used to calculate statistical significance using GraphPad Prism software. P values: *P < 0.05, **0.01 < P < 0.05, ***P < 0.001.

## Supplementary information

Supplementary figures and legend

## References

[1] Ho, I. C., Hodge, M. R., Rooney, J. W. & Glimcher, L. H. The proto-oncogene c-maf is responsible for tissue-specific expression of interleukin-4. *Cell* **85**, 973–983, doi:S0092-8674(00)81299-4 [pii] (1996). 10.1016/s0092-8674(00)81299-4 8674125

[2] Ho IC, Lo D, Glimcher LH (1998). c-maf promotes T helper cell type 2 (Th2) and attenuates Th1 differentiation by both interleukin 4-dependent and -independent mechanisms. J Exp Med.

[3] Rutz S (2011). Transcription factor c-Maf mediates the TGF-beta-dependent suppression of IL-22 production in T(H)17 cells. Nat Immunol.

[4] Xu J (2009). c-Maf regulates IL-10 expression during Th17 polarization. J Immunol.

[5] Kroenke MA (2012). Bcl6 and Maf cooperate to instruct human follicular helper CD4 T cell differentiation. J Immunol.

[6] Gabrysova L (2018). c-Maf controls immune responses by regulating disease-specific gene networks and repressing IL-2 in CD4(+) T cells. Nat Immunol.

[7] Yu JS (2017). Differentiation of IL-17-Producing Invariant Natural Killer T Cells Requires Expression of the Transcription Factor c-Maf. Frontiers in immunology.

[8] Giordano M (2015). Molecular profiling of CD8 T cells in autochthonous melanoma identifies Maf as driver of exhaustion. EMBO J.

[9] Apetoh L (2010). The aryl hydrocarbon receptor interacts with c-Maf to promote the differentiation of type 1 regulatory T cells induced by IL-27. Nat Immunol.

[10] Lochner M (2008). *In vivo* equilibrium of proinflammatory IL-17+ and regulatory IL-10+ Foxp3+ RORgamma t+ T cells. J Exp Med.

[11] Sefik E (2015). MUCOSAL IMMUNOLOGY. Individual intestinal symbionts induce a distinct population of RORgamma(+) regulatory T cells. Science.

[12] Ohnmacht C (2015). MUCOSAL IMMUNOLOGY. The microbiota regulates type 2 immunity through RORgammat(+) T cells. Science.

[13] Yang BH (2016). Foxp3(+) T cells expressing RORgammat represent a stable regulatory T-cell effector lineage with enhanced suppressive capacity during intestinal inflammation. Mucosal Immunol.

[14] Kim KS (2016). Dietary antigens limit mucosal immunity by inducing regulatory T cells in the small intestine. Science.

[15] Xu M (2018). c-MAF-dependent regulatory T cells mediate immunological tolerance to a gut pathobiont. Nature.

[16] Wheaton JD, Yeh CH, Ciofani M (2017). Cutting Edge: c-Maf Is Required for Regulatory T Cells To Adopt RORgammat(+) and Follicular Phenotypes. J Immunol.

[17] Aschenbrenner D (2018). An immunoregulatory and tissue-residency program modulated by c-MAF in human TH17 cells. Nat Immunol.

[18] Mottet C, Uhlig HH, Powrie F (2003). Cutting edge: cure of colitis by CD4+CD25+regulatory T cells. J Immunol.

[19] Moyat M, Velin D (2014). Immune responses to Helicobacter pylori infection. World J Gastroenterol.

[20] Velin D, Bachmann D, Bouzourene H, Michetti P (2008). Reduction of Helicobacter infection in IL-10-/- mice is dependent on CD4+T cells but not on mast cells. Helicobacter.

[21] Velin D (2009). Interleukin-17 is a critical mediator of vaccine-induced reduction of Helicobacter infection in the mouse model. Gastroenterology.

[22] Moyat M (2017). IL-22-induced antimicrobial peptides are key determinants of mucosal vaccine-induced protection against H. pylori in mice. Mucosal Immunol.

[23] Kim, J. I., Ho, I. C., Grusby, M. J. & Glimcher, L. H. The transcription factor c-Maf controls the production of interleukin-4 but not other Th2 cytokines. *Immunity* **10**, 745–751, doi:S1074-7613(00)80073-4 [pii] (1999). 10.1016/s1074-7613(00)80073-4 10403649

[24] Fuss IJ (1996). Disparate CD4+ lamina propria (LP) lymphokine secretion profiles in inflammatory bowel disease. Crohn’s disease LP cells manifest increased secretion of IFN-gamma, whereas ulcerative colitis LP cells manifest increased secretion of IL-5. J Immunol.

[25] Huber S (2011). Th17 cells express interleukin-10 receptor and are controlled by Foxp3(-) and Foxp3+ regulatory CD4+ T cells in an interleukin-10-dependent manner. Immunity.

[26] Rubtsov YP (2008). Regulatory T cell-derived interleukin-10 limits inflammation at environmental interfaces. Immunity.

[27] Chaudhry A (2011). Interleukin-10 signaling in regulatory T cells is required for suppression of Th17 cell-mediated inflammation. Immunity.

[28] Roers A (2004). T cell-specific inactivation of the interleukin 10 gene in mice results in enhanced T cell responses but normal innate responses to lipopolysaccharide or skin irritation. J Exp Med.

[29] Kuhn R, Lohler J, Rennick D, Rajewsky K, Muller W (1993). Interleukin-10-deficient mice develop chronic enterocolitis. Cell.

[30] Solomon BD, Hsieh CS (2016). Antigen-Specific Development of Mucosal Foxp3+RORgammat+T Cells from Regulatory T Cell Precursors. J Immunol.

[31] Sawada S, Scarborough JD, Killeen N, Littman DR (1994). A lineage-specific transcriptional silencer regulates CD4 gene expression during T lymphocyte development. Cell.

[32] Wende H (2012). The transcription factor c-Maf controls touch receptor development and function. Science.

[33] Velin D, Bachmann D, Bouzourene H, Michetti P (2005). Mast cells are critical mediators of vaccine-induced Helicobacter clearance in the mouse model. Gastroenterology.

