# Supplementary material for: Maf deficiency in T cells dysregulates Treg - TH17 balance leading to spontaneous colitis
Source: Sci Rep. 2019 Apr 16;9:6135. doi: 10.1038/s41598-019-42486-2 (PMC6468010; doi:10.1038/s41598-019-42486-2)

Maf deficiency in T cells dysregulates T_{reg} - T_H17 balance leading to spontaneous colitis

Claire Imbratta[1], Marine M Leblond[1], Hanifa Bouzourène[2], Daniel E Speiser[1], Dominique Velin[3], Grégory Verdeil[1*]

Supplementary Figure 1.

A Colon length of *Maf*$^{f/f}$ and *Maf*$^{\Delta Tcells}$ mice (n>12 per group). Haematoxylin and eosin-stained sections of liver (**B**) and kidney (**C**) of *Maf*$^{f/f}$ (upper) and *Maf*$^{\Delta Tcells}$ (bottom) mice (scale bar 100µm and 50 µm for 20X and 40X respectively). **D** Weight and volume of kidneys from *Maf*$^{f/f}$ and *Maf*$^{\Delta Tcells}$. **E** Histological scores (from 0 to 6) of microscopic changes in the colon from *Maf*$^{f/f}$ and *Maf*$^{\Delta Tcells}$ antibiotics treated mice (n≥ 4 per group). **F** Representative pictures of colons from *Maf*$^{f/f}$ and *Maf*$^{\Delta Tcells}$ mice treated with antibiotics at low (left) and high (right) magnification (bars represent 200 µm and 50µm respectively). All mice were over 20 weeks old. Each symbol represents an individual mouse or organ. All graphs indicate means.

Supplementary Figure 2.

$CD4^+$ T cells isolated from splenocytes of WT and *Maf*$^{\Delta Tcells}$ mice were cultured for 5 days in T_H1 (IL-12 + anti-IL-4), T_H2 (IL-4 + anti-IFN-γ), T_{reg} (TGF-β +IL-2) or T_H17 (TGF-β +IL-6) polarizing conditions. **A** Gating strategy used before gating on master transcription factors as shown in figure 2. **B** Representative dot plots of master transcription factors (TBET, GATA3, FOXP3 and RORγt) for each polarizing conditions in CD4 T cells obtained from WT mice factor. **C** Supernatants of each culture were recovered at the end of the experiment. Level of the indicated cytokines was analysed usind MSD technology. Data are representative of 2 independent experiments with at least 2 mice per group. As IL-4 is used in the T_H2 culture condition we do not show IL-4 level for this condition. All graphs indicate means. Error bars display SEM.

Supplementary Figure 3.

A Absolute cell numbers of RORγt^{-} T_{reg}, RORγt^{+} T_{reg} and T_H17 cells from colons of WT and *Maf*^{ΔTcells} mice (n>10 per group). **B** Absolute cell numbers of RORγt^{-} T_{reg}, RORγt^{+} T_{reg} and T_H17 cells from colons of WT and *Maf*^{ΔTreg} mice (n>7 per group). All mice were over 20 weeks old. Each symbol represents an individual mouse. All graphs indicate means. **C** Proportion of CD4+ GATA3+ T cells (Th2), IL-10+ RORγt- FOPXP3- CD4+ T cells (Tr1), RORγt- FOPXP3+ CD4+ T cells (RORγt- Treg), RORγt+ FOPXP3+ CD4+ T cells (RORγt+ Treg) and RORγt+ FOPXP3- CD4+ T cells (Th17) among CD4+ T cells in the colon of 4 to 8 *Maf*$^{f/f}$ mice and 4 to 8 *Maf*^{ΔTcells} mice.

Supplementary Figure 4.

Quantitative RT–PCR of stomachs from non-infected WT (n= 6) and *Maf*^{ΔTcells} (n= 6) mice and *Helicobacter pylori* infected WT (n=6) and *Maf*^{ΔTcells} (n= 5) mice for the indicated transcripts. Gene expression levels were normalized to *gapdh*. Each symbol represents an individual mouse. Data are representative of at least 2 independent experiments with at least 2 mice per group.

Supplementary Figure 1.

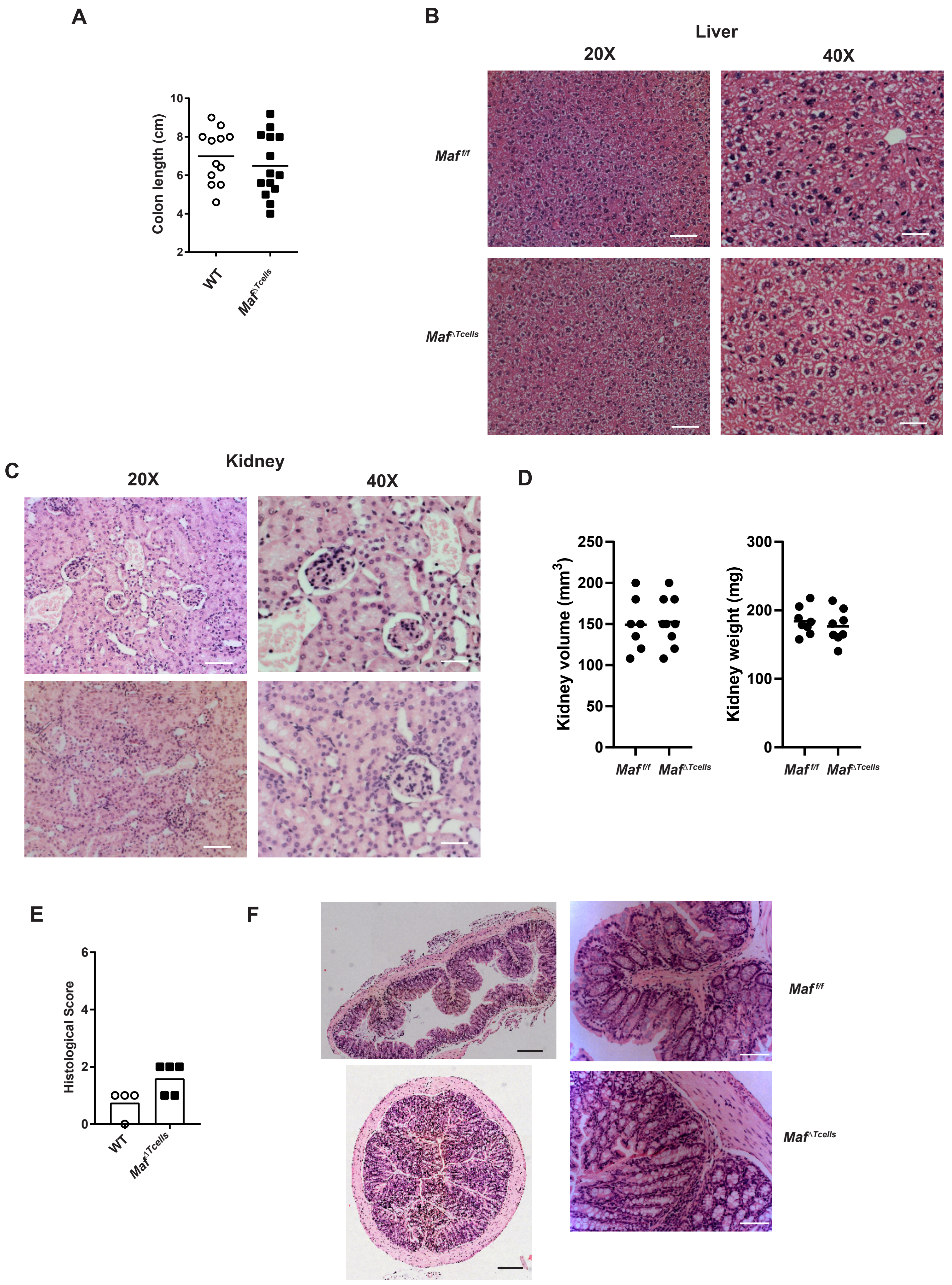

Supplementary figure 2

A

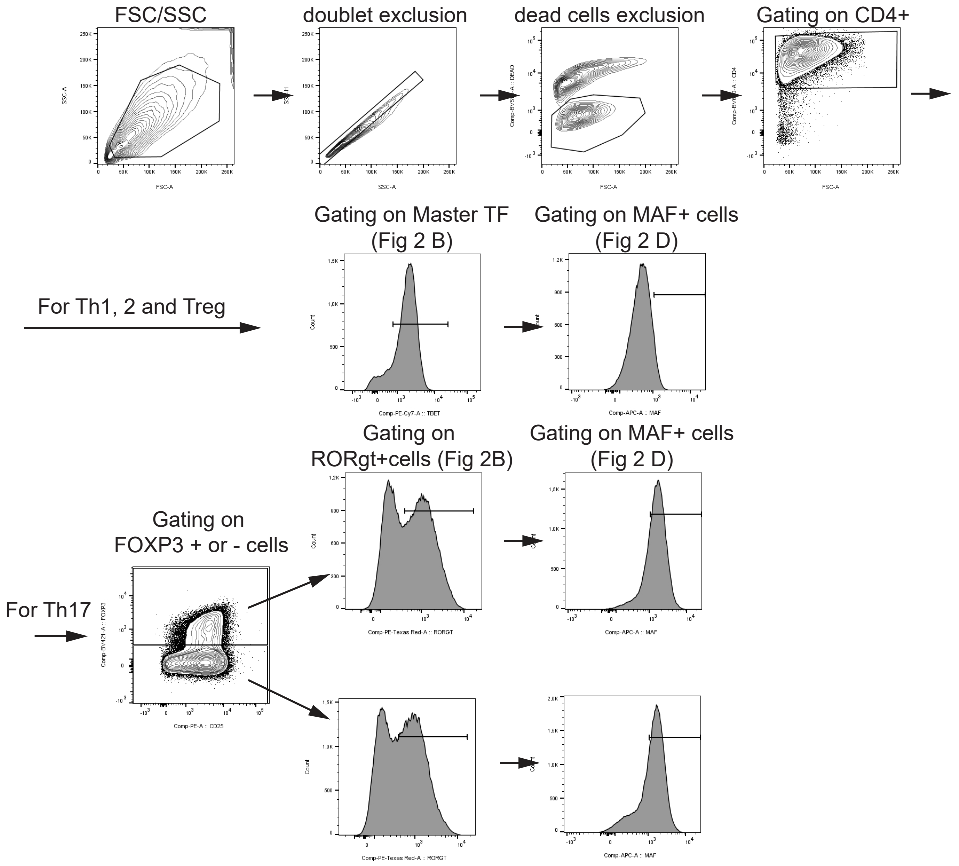

B

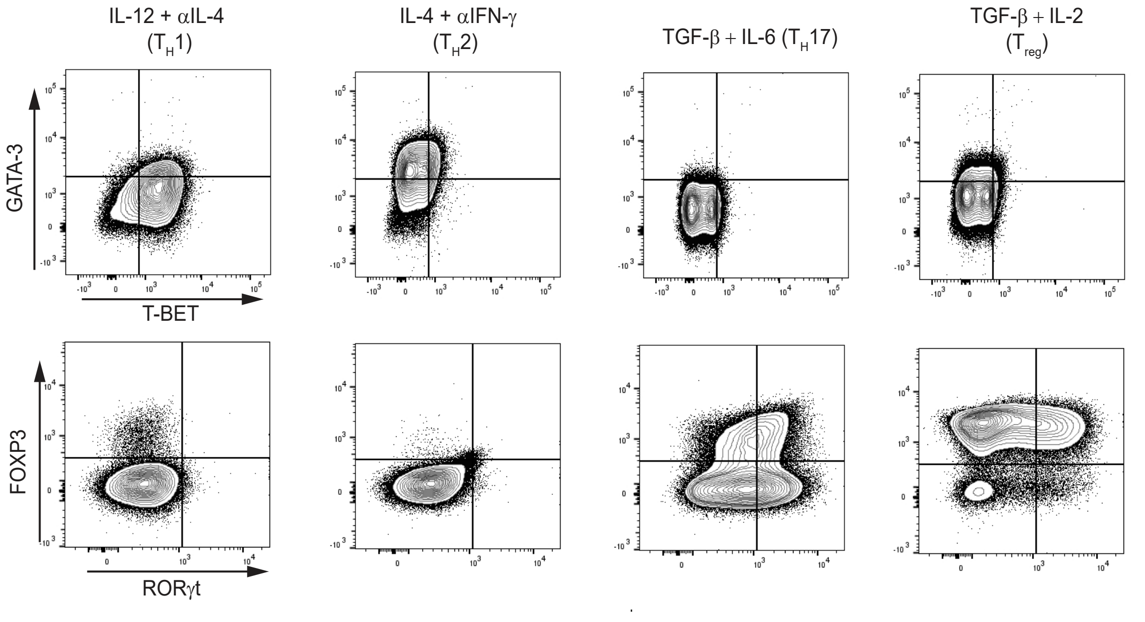

C

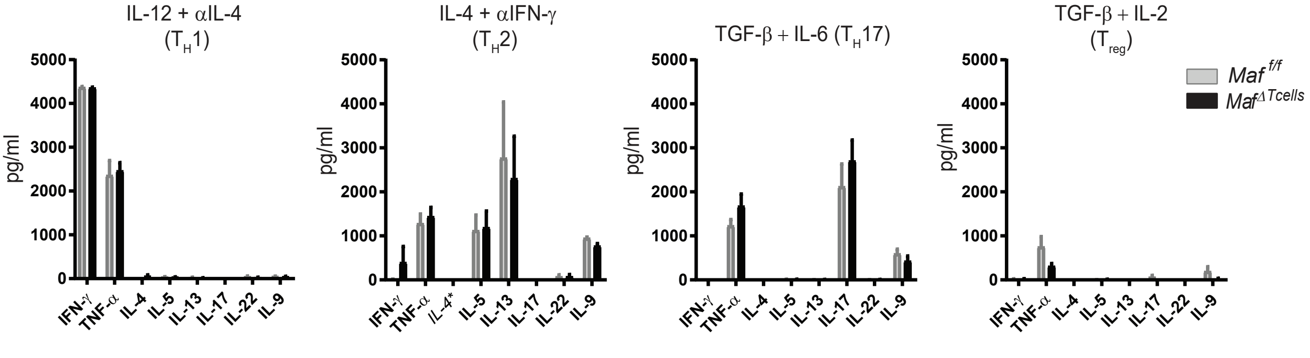

Supplementary Figure 3.

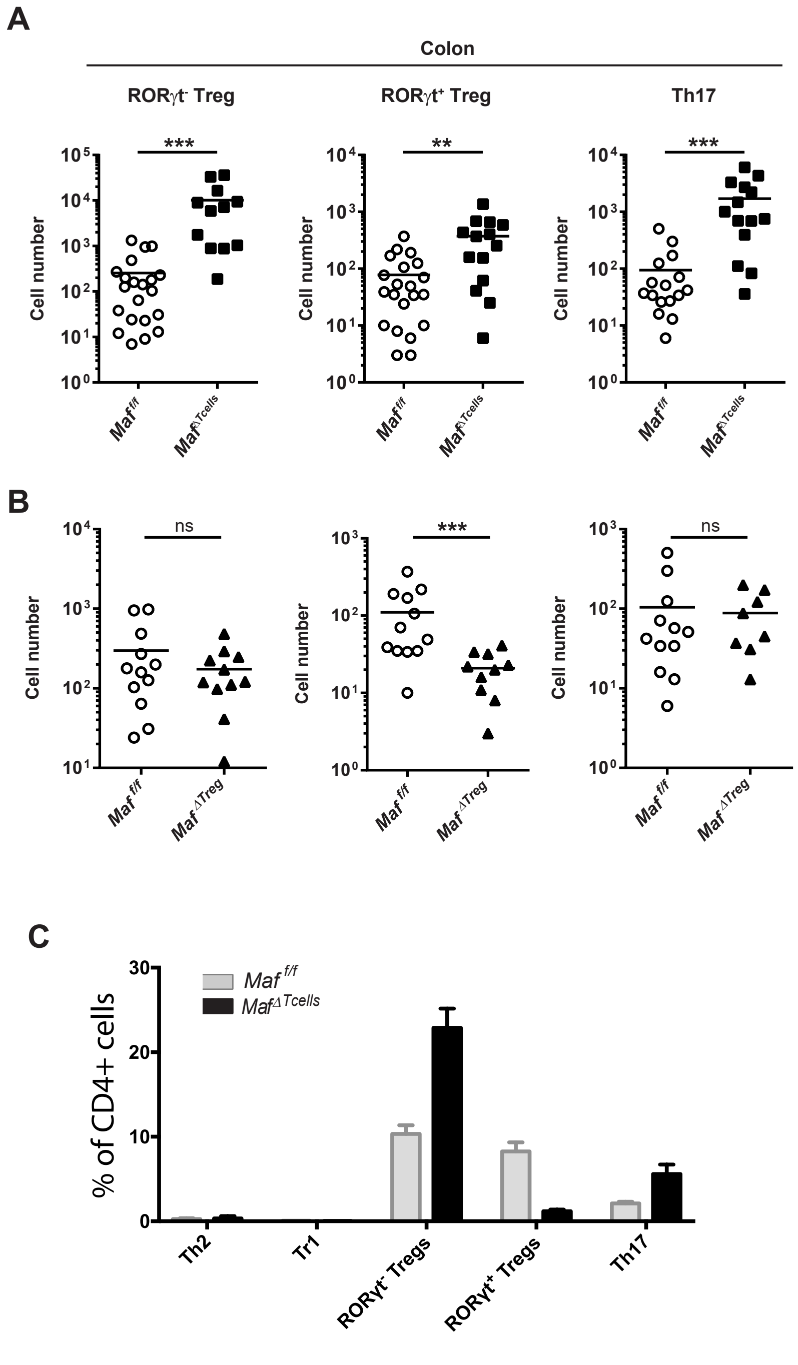

Supplementary Figure 4.

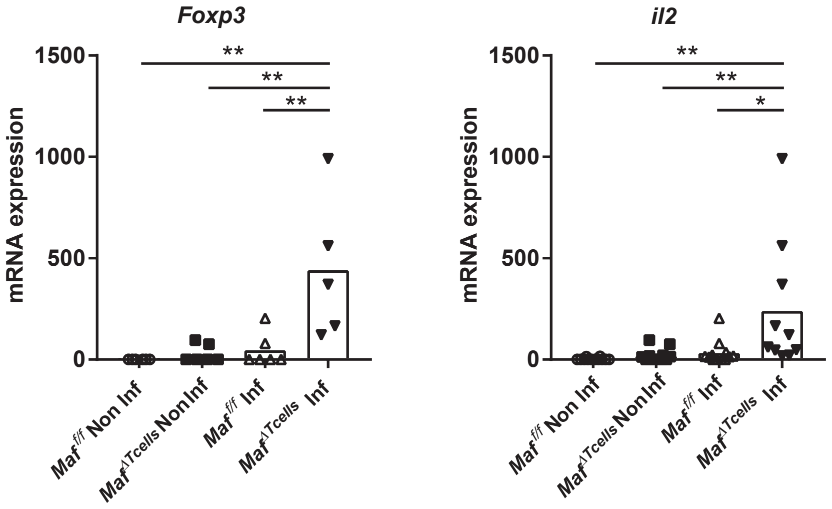

Supplement: Supplementary file 1 — Supplementary figures and legend [file 41598_2019_42486_MOESM1_ESM.pdf]
